# Dose-compatible grating-based phase-contrast mammography on mastectomy specimens using a compact synchrotron source

**DOI:** 10.1038/s41598-018-33628-z

**Published:** 2018-10-24

**Authors:** Elena Eggl, Susanne Grandl, Anikό Sztrόkay-Gaul, Martin Dierolf, Christoph Jud, Lisa Heck, Karin Burger, Benedikt Günther, Klaus Achterhold, Doris Mayr, Jan J. Wilkens, Sigrid D. Auweter, Bernhard Gleich, Karin Hellerhoff, Maximilian F. Reiser, Franz Pfeiffer, Julia Herzen

**Affiliations:** 1 Chair of Biomedical Physics, Department of Physics, Technical University of Munich, James-Franck-Straße 1, 85748 Garching, Germany; 2 Munich School of BioEngineering, Technical University of Munich, Boltzmannstraße 11, 85748 Garching, Germany; 3 Institute for Clinical Radiology, Ludwig-Maximilians-University Hospital Munich, Marchioninistraße 15, 81377 München, Germany; 4 Max-Planck-Institute for Quantum Optics, Hans-Kopfermann-Straße 1, 85748 Garching, Germany; 5 Institute of Pathology, Ludwig-Maximilians-University München, Thalkirchner Straße 36, 80337 München, Germany; 6 Department of Diagnostic and Interventional Radiology, Klinikum rechts der Isar, Technical University of Munich, Ismaninger Straße 22, 81675 München, Germany; 7 Department of Radiation Oncology, Klinikum rechts der Isar, Technical University of Munich, Ismaninger Straße 22, 81675 München, Germany

## Abstract

With the introduction of screening mammography, the mortality rate of breast cancer has been reduced throughout the last decades. However, many women undergo unnecessary subsequent examinations due to inconclusive diagnoses from mammography. Two pathways appear especially promising to reduce the number of false-positive diagnoses. In a clinical study, mammography using synchrotron radiation was able to clarify the diagnosis in the majority of inconclusive cases. The second highly valued approach focuses on the application of phase-sensitive techniques such as grating-based phase-contrast and dark-field imaging. Feasibility studies have demonstrated a promising enhancement of diagnostic content, but suffer from dose concerns. Here we present dose-compatible grating-based phase-contrast and dark-field images as well as conventional absorption images acquired with monochromatic x-rays from a compact synchrotron source based on inverse Compton scattering. Images of freshly dissected mastectomy specimens show improved diagnostic content over *ex-vivo* clinical mammography images at lower or equal dose. We demonstrate increased contrast-to-noise ratio for monochromatic over clinical images for a well-defined phantom. Compact synchrotron sources could potentially serve as a clinical second level examination.

## Introduction

Mammography is an invaluable clinical tool for the early detection of breast cancer. However, the inherently low contrast in absorption x-ray imaging for soft tissue compromises the diagnostic performance, especially in the case of dense breasts. The radiation sensitivity of breast tissue limits the allowed dose^1^. Recent literature reports a sensitivity between 69% and 94% and a specificity between 78% and 95% for digital mammography, depending on patient age and breast density^2^. The positive predictive value is between 7% and 13% in screening examinations^3^. These numbers explain why the field of mammography is vastly under research and a non-invasive second-level examination besides ultrasonography is needed to clarify questionable or suspicious findings and avoid unnecessary invasive procedures such as biopsies.

A strongly investigated topic is mammography with synchrotron radiation. The x-ray energy can be optimized for the best ratio of contrast to dose, thus eliminating the x-ray photons that do not give sufficient contrast (above the ideal energy) or those that deposit too much dose (below the ideal energy). A large clinical study at the SYRMEP beamline of the synchrotron Elettra (Trieste, Italy) has aimed at the patient group with inconclusive diagnosis after mammography and ultrasonography. The combination of absorption contrast and edge-enhancement resulting from phase shifts and subsequent free-space propagation enabled higher relative visibilities of abnormalities and the number of true-negative findings was significantly increased, while the dose level was lower or comparable to conventional mammography^4–6^. However, mammography with synchrotron radiation has the disadvantages of limited availability, remoteness with respect to clinics, and high costs.

A different approach to access phase information is x-ray imaging with a grating interferometer, which simultaneously provides attenuation, differential phase and dark-field signals^7,8^. Several studies have already successfully shown the application of phase-contrast imaging for mammography^9–11^ and especially for the classification of microcalcifications^12^. The dark-field signal is related to small-angle scattering and has been shown to improve the visibility of microcalcifications^13^ or could even help to distinguish between different types of calcifications^14^. Various studies carried out with dissected cancerous breasts at conventional x-ray tubes have been conducted, showing significantly higher diagnostic content provided by the multimodal images^15–17^. However, initial investigations of this technique were performed with applied radiation doses far above radiological guidelines. Only recently, dose-compatible phase-contrast mammography images were presented^18^. In addition, the feasibility of installing a grating interferometer into a conventional digital mammography unit was successfully demonstrated^19,20^. Challenges are the low sensitivity of the interferometer due to the limited distance between the gratings, and dose compatibility.

In order to combine the advantages and avoid some disadvantages of the two approaches, we suggest to perform grating-based multimodal mammography at a compact synchrotron source based on inverse Compton scattering. These recently developed x-ray sources are compact enough to fit into regular laboratories^21^. They offer a monochromatic x-ray beam that is tunable in energy and partially coherent, allowing to obtain increased diagnostic content from grating-based phase-contrast and dark-field images while achieving equal image quality at lower dose compared to the aforementioned laboratory setups. In addition, monochromatic conventional absorption-based imaging or propagation-based phase-contrast imaging as done at SYRMEP is possible. Since the spatial and financial requirements of inverse Compton sources are relatively small compared to large-scale synchrotrons, the installation at medical centers appears feasible. The Munich Compact Light Source (MuCLS, Lyncean Technologies, USA) is the first commercial installation of an inverse Compton compact synchrotron source^22^.

Here, we present both absorption-only and grating-based multimodal images of freshly dissected cancerous mastectomy specimens acquired at the MuCLS at lower or equal dose compared to state-of-the-art clinical images. Furthermore, a dose study and analysis of the contrast-to-noise-ratio on a well defined sample, a mammographic accreditation phantom, was conducted. In addition, we present a comparison of the spatial resolution for experimental and clinical images. We believe that compact synchrotron sources like the MuCLS have great potential to bring benefits to clinical imaging, in particular for mammography, but also other fields, like coronary angiography^23^, could profit from the monochromatic, tunable x-ray beam.

## Results

Four freshly dissected mastectomy specimens and a mammographic accreditation phantom were investigated with *ex-vivo* clinical mammography (cevAC-Mx) and both conventional absorption and grating-based multimodal monochromatic experimental mammography. Abbreviations used for the different contrast modalities in the following are given in Table 1. An overview of the CNR and the spatial resolution analysis is presented in Tables 2 and 3, respectively. The tumor characteristics for each sample are summarized in Table 4. The applied mean glandular dose (MGD) and the exposure times are also given in Table 4 for each sample and imaging modality.

**Table 1:** Contrast modality abbreviations.

mAC-Mx	monochromatic absorption-contrast mammography
mgbAC-Mx	monochromatic grating-based absorption-contrast mammography
mgbDPC-Mx	monochromatic grating-based differential phase-contrast mammography
mgbDFC-Mx	monochromatic grating-based dark-field-contrast mammography
cevAC-Mx	clinical *ex-vivo* absorption-contrast mammography
civAC-Mx	clinical *in-vivo* absorption-contrast mammography

**Table 2:** CNR calculated for dose study with mammographic accreditation phantom.

Modality	MGD [mGy]	Fibers		Calcifications		Tumor Masses	
		1	4	1	3	1	5
cevAC-Mx	2.0	2.51	1.99	36.84	13.89	5.92	0.53
mAC-Mx	1.0	3.16	0.11	30.86	11.94	8.64	1.50
mAC-Mx	1.6	3.51	1.61	38.47	15.60	10.70	2.73
mAC-Mx	2.0	4.71	1.39	44.11	16.85	12.19	2.67
mgbAC-Mx	1.8	4.46	0.15	25.32	13.25	7.14	0.89
mgbDFC-Mx	1.8	0.65	0.16	6.50	10.42	15.00	9.59
mgbAC-Mx	0.7	2.17	0.09	13.87	5.93	2.73	0.30
mgbDFC-Mx	0.7	1.25	2.55	3.59	7.77	11.84	5.58

**Table 3:** Resolution calculated from power spectrum analysis.

Sample	cevAC-Mx [LP/mm]	mAC-Mx [LP/mm]	mgbAC-Mx [LP/mm]	mgbDFC-Mx [LP/mm]
Phantom	3.70 ± 0.26	3.81 ± 0.11	3.55 ± 0.26	3.90 ± 0.74
I	2.52 ± 0.68	2.77 ± 0.41	3.03 ± 0.77	3.87 ± 0.70
II	1.42 ± 0.17	—	3.44 ± 0.16	5.30 ± 0.53
III	3.23 ± 0.68	3.37 ± 0.61	3.49 ± 0.07	3.56 ± 0.61
VI	2.94 ± 1.43	3.49 ± 0.66	—	—

**Table 4:** Parameters and information for each specimen.

Specimen	I	II	II	IV	Phantom
**Tumor characteristics as verified by histopathology**					
Histological diagnosis	multicentric lobular invasive carcinoma, G2, and lobular carcinoma *in situ*	recurrent, invasive carcinoma of no specific type (NST, formerly invasive ductal), G3	invasive carcinoma of no specific type (NST, formerly invasive ductal), G2, and adjacent intraductal carcinoma (DCIS)	bifocal invasive carcinoma of no specific type (NST, formerly invasive ductal), G1, and intraductal carcinoma (DCIS)	
Max. tumor diameter	51 mm	47 mm	5 mm	25 mm	
**Sample position for** *ex-vivo* **mammography**					
Orientation	AP	AP	AP	CC	—
Compressed thickness [cm]	4.0	6.0	4.5	6.0	4.5
**Clinical acquisition parameters**					
X-ray tube settings	27 kVp (W/Rh)	35 kVp (W/Ag)	30 kVp (W/Rh)	39 kVp (W/Ag)	28 kVp (W/Rh)
	72 mAs	92 mAs	100 mAs	23 mAs	200 mAs
MGD civAC-Mx [mGy]	1.3	3.8	2.9	1.3	—
MGD cevAC-Mx [mGy]	0.9	2.2	1.4	1.1	2.0
**Acquisition parameters MuCLS**					
Energy	25 keV	25 keV	25 keV	25 keV	25 keV
MGD mAC-Mx [mGy]	—	—	0.3	0.4	1.0–2.0
Exposure times [s]	—	—	3	3	10–20
MGD mgb-Mx [mGy]	0.8	0.9	0.9	—	0.7–1.8
Exposure times [s]	7	14	11	—	7–18
Stitching	4 × 4	5 × 5	5 × 5	4 × 4	2 × 2

### Improved delineation of tumor lesions in phase-contrast image

**Figure 1:**
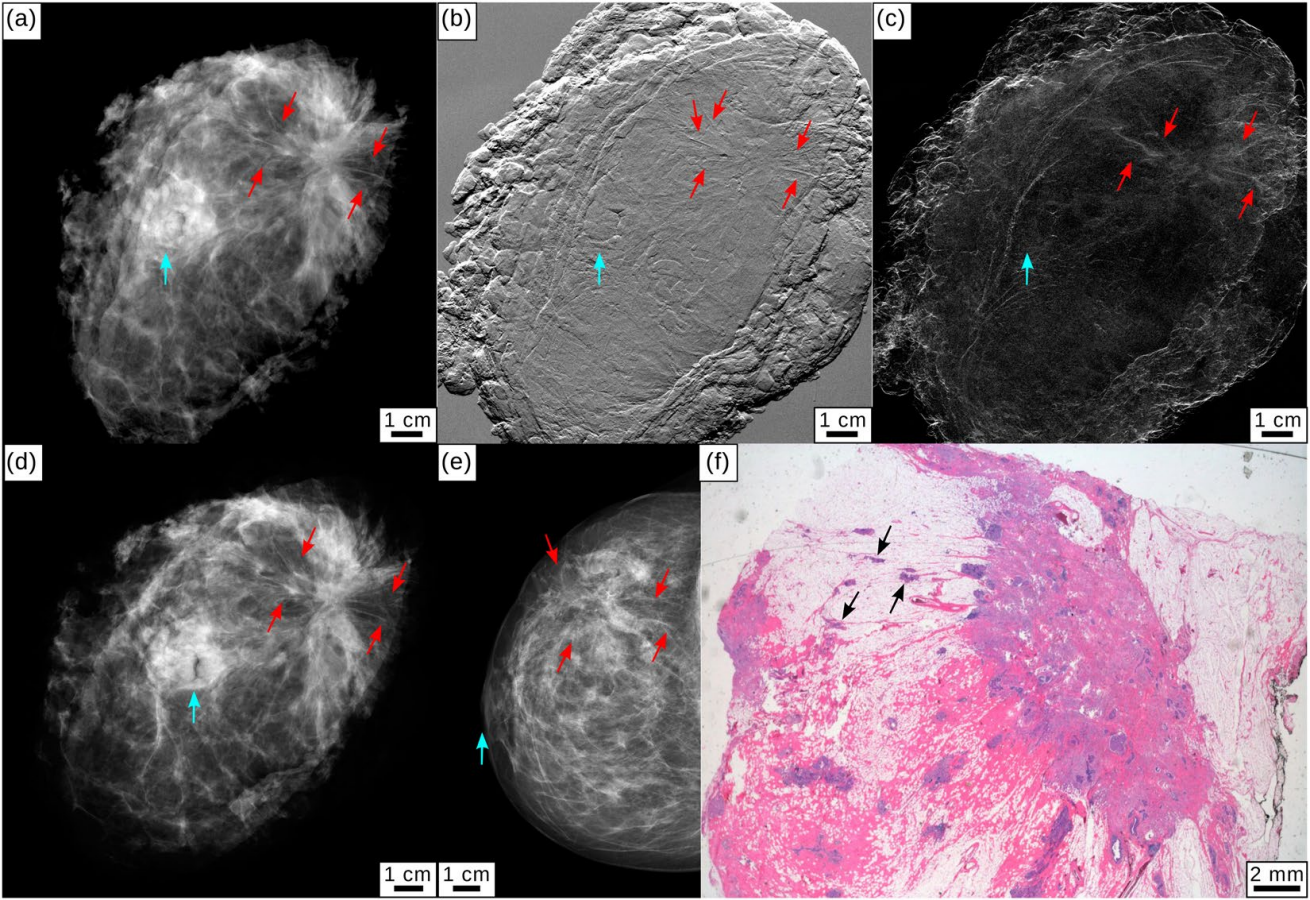
Clinical mammography and monochromatic grating-based multimodal mammography for specimen I. (**a**) Monochromatic grating-based absorption-contrast (mgbAC-Mx), (**b**) differential phase-contrast (mgbDPC-Mx), and (**c**) dark-field contrast (mgbDFC-Mx) mammography. (**d**) Clinical *ex-vivo* absorption-contrast mammography (cevAC-Mx) in anteroposterior position. (**e**) Clinical *in-vivo* absorption-contrast mammography (civAC-Mx) of specimen I in cranio-caudal position. Tumorous lesions are indicated by red arrows, the mamilla is indicated by a light blue arrow. All images were scaled for maximum detail visibility. (**f**) Histopathology of the mastectomy sample showing the existence of tumorous lesions (black arrows).

Specimen I presented with a palpable mass in the right breast and skin retraction. Conventional *in-vivo* mammography (civAC-Mx) showed an asymmetry in the respective region (Fig. 1(e)). Ultrasound revealed inhomogeneities and several hypoechogenic lesions. Additional dynamic MRI showed an extensive infiltration with tumor branches extending close to the pectoralis muscle. Histopathology of the mastectomy specimen revealed a multicentric, invasive lobular carcinoma (G2) with extensive manifestations of a lobular carcinoma *in situ*.

Figure 1(a–d) show monochromatic grating-based absorption-contrast (mgbAC-Mx), differential phase-contrast (mgbDPC-Mx), dark-field contrast (mgbDFC-Mx) and *ex-vivo* clinical absorption-contrast (cevAC-Mx) mammography images of the mastectomy specimen from specimen I, measured in anteroposterior (AP) orientation. The mgbAC-Mx (a) provides improved delineation of tumor lesions (marked by red arrows) over the cevAC-Mx (d) and civAC-Mx (e) images. The mgbDPC-Mx (b) clearly shows fine tumor branches originating from the tumor and perfusing the surrounding tissue to both sides of the carcinoma. To a reduced extent, the tumor branches are also visible in mgbDFC-Mx (c). The histopathologic analysis in hematoxylin-eosin (H&E) staining proved the existence of the tumor spiculae originating from the main tumor (black arrows, (e)).

Specimen II had a history of breast cancer. While aftercare mammography and ultrasound showed a slight, ill-defined density in the scar area but no circumscribed lesion, dynamic MRI of the right breast revealed an extensive carcinoma. Histology of the mastectomy sample showed nodular infiltrations of a poorly differentiated (G3) invasive breast cancer of no special type.

**Figure 2:**
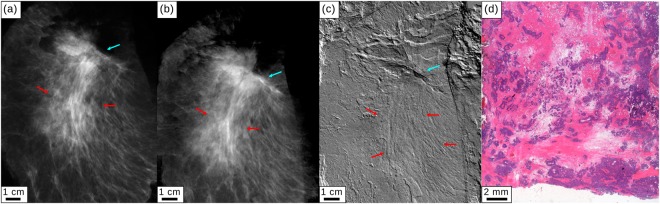
Clinical mammography and monochromatic grating-based absorption-contrast and differential phase-contrast mammography for specimen II. (**a**) Clinical *ex-vivo* absorption-contrast mammography (cevAC-Mx). (**b**) Monochromatic grating-based absorption-contrast (mgbAC-Mx) and (**c**) monochromatic grating-based differential phase-contrast (mgbDPC-Mx) mammography. Red arrows indicate the tumorous region with spiculae visible especially in the mgbDPC-Mx (**c**). The cyan arrow points to the mamilla. All images were scaled for maximum detail visibility. (**d**) Histopathology of the mastectomy sample showing the scar tissue (pink) being infiltrated by tumor cells (purple).

Figure 2(a–c) show cevAC-Mx, mgbAC-Mx, and mgbDPC-Mx measured in an AP orientation of the specimen. While the differentiation of tumor and scar tissue is difficult in the AC images, the mgbDPC-Mx (c) depicts the tumor spiculae (red arrows) as verified by histopathology (d). The H&E stained histology image shows the infiltration of the scar tissue (pink) with tumorous cells (purple).

### Detection of microcalcifications at reduced dose

In Specimen III, civAC-Mx revealed a cluster of microcalcifications in the retromamillary area. Ultrasound found a hypoechoic lesion and MRI showed a corresponding suspicious mass with early contrast enhancement. Histology of the mastectomy sample revealed an invasive carcinoma of no special type, G2, and an adjacent intraductal carcinoma (DCIS), with comedonecrosis and extensive microcalcifications.

**Figure 3:**
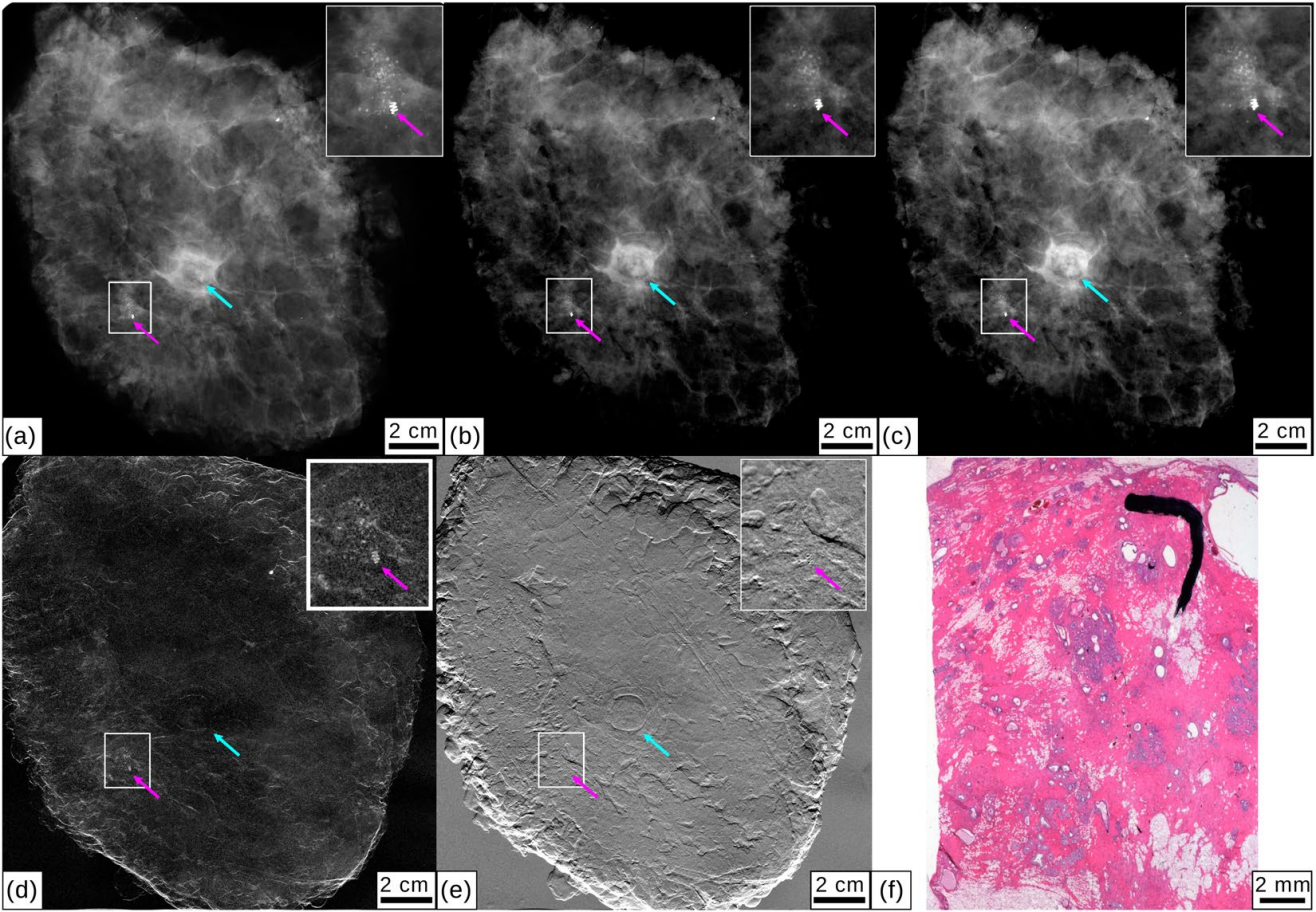
Clinical mammography and monochromatic absorption-contrast and grating-based multimodal mammography for specimen III. (**a**) Clinical *ex-vivo* absorption-contrast mammography (cevAC-Mx). (**b**) Monochromatic absorption-contrast mammography (mAC-Mx). (**c**) Monochromatic grating-based absorption-contrast mammography (mgbAC-Mx), (**d**) dark-field mammography (mgbDFC-Mx) and (**e**) differential phase-contrast mammography (mgbDPC-Mx). Inlets show a calcification cluster that had previously been marked. The clip marker is highlighted with a magenta arrow, a light blue arrow indicates the mamilla. All images were scaled for maximum detail visibility. (**f**) Histopathology of the mastectomy sample showing microcalcifications.

Figure 3(a–e) display cevAC-Mx, mAC-Mx, mgbAC-Mx, mgbDPC-Mx and mgbDFC-Mx measured in AP orientation. A calcification cluster that had preoperatively been clip-marked (magenta arrows) is shown in magnification in the small inlets. The mAC-Mx image (b) equally shows the extent of the microcalcification cluster compared to cevAC-Mx (a), at significantly lower dose (0.3 vs. 1.4 mGy). The existence of the microcalcification cluster was verified by histopathology (f). The invasive carcinoma revealed by histopathology could not clearly be identified by any of the imaging modalities, including civAC-Mx.

Specimen IV was diagnosed with three microcalcification clusters in civAC-Mx, two of which were partially extracted in a biopsy. Histology of the mastectomy sample revealed a bifocal, well differentiated (G1) breast cancer of no special type, with an extensive intraductal carcinoma (DCIS) and microcalcifications.

**Figure 4:**
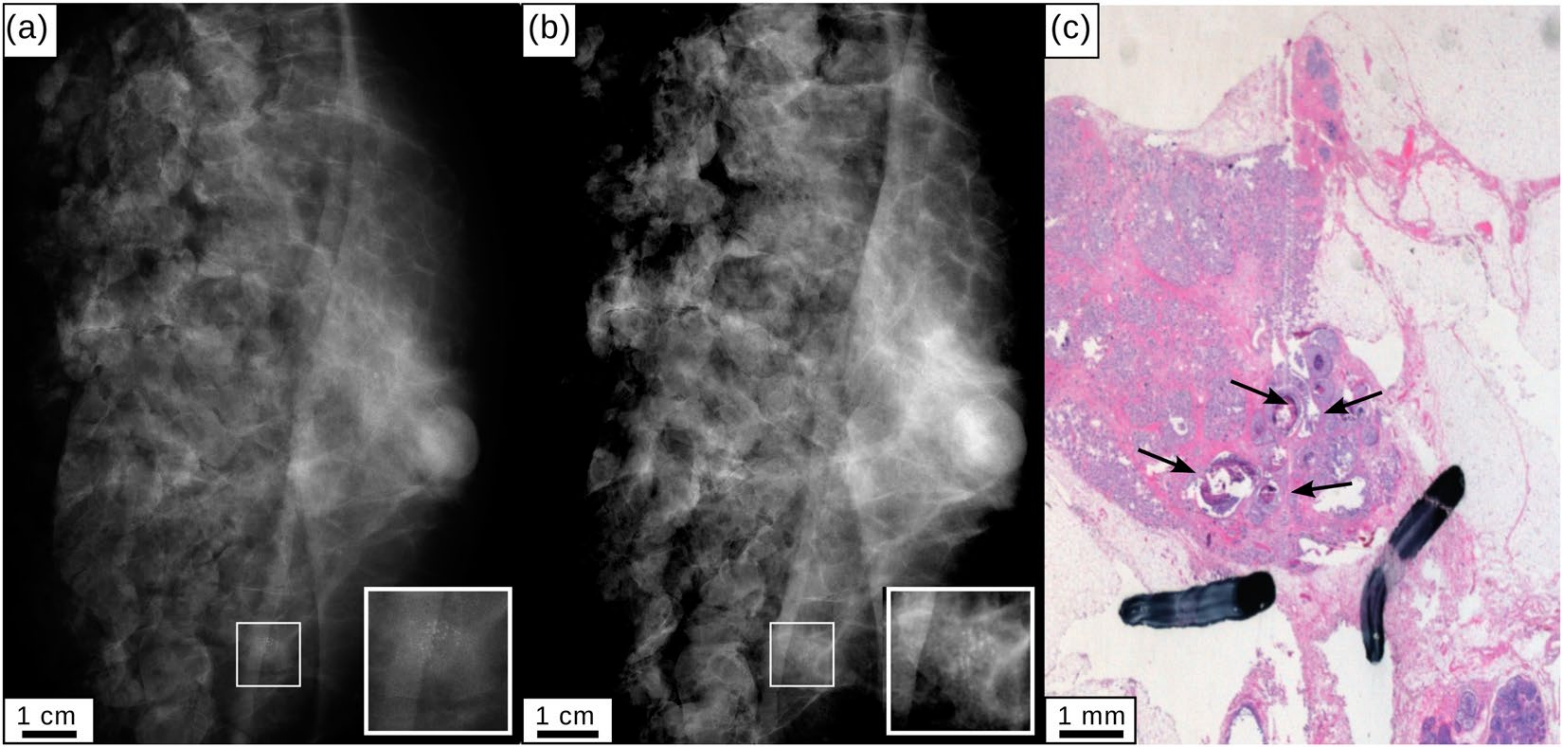
Clinical mammography and monochromatic absorption-contrast mammography for specimen IV. (**a**) Clinical mammography (cevAC-Mx). (**b**) Monochromatic absorption-contrast mammography (mAC-Mx). Inlets show a calcification cluster. All images were scaled for maximum detail visibility. (**c**) Histopathology of the mastectomy specimen showing extensive microcalcifications (black arrows).

Figure 4(a,b) present cevAC-Mx and mAC-Mx measured in a cranio-caudal position. Both images reveal a microcalcification cluster to equal extent, with significantly lower dose for the mAC-Mx (0.4 vs. 1.1 mGy). The histopathologic work-up (c) confirms the existence of microcalcifications. The bifocal carcinoma revealed by histopathology is obscured by the surgical margins of the mastectomy sample in images (a) and (b).

### Dose-study with a mammographic accreditation phantom

**Figure 5:**
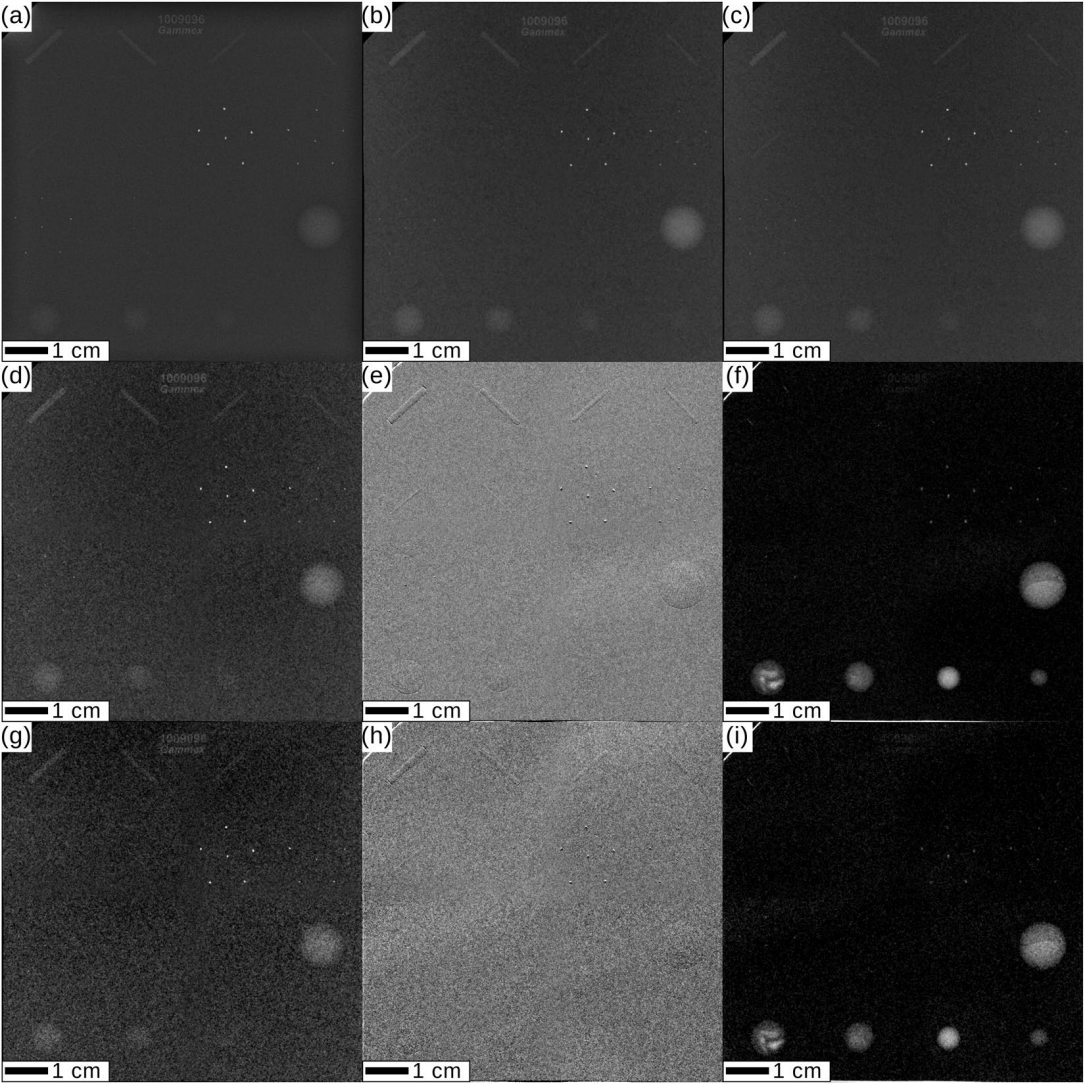
Dose study for the mammographic accreditation phantom. ACR guidelines require for a minimum of four fibrils, three groups of microcalcifications, and three tumor masses to be resolved. (**a**) Clinical mammography cevAC-Mx acquired at 2.0 mGy mean glandular dose (MGD). (**b**,**c**) Monochromatic absorption-contrast mammography (mAC-Mx) acquired at 2.0 mGy MGD (**b**) and 1.6 mGy MGD (**c**). (**d**–**f**) Monochromatic grating-based absorption-contrast (mbgAC-Mx) (**d**), differential phase-contrast (mgbDPC-Mx) (**e**) and dark-field-contrast (mgbDFC-Mx) (**f**) mammography acquired at 1.8 mGy MGD. (**g**–**i**) mgbAC-Mx (**g**), mgbDPC-Mx (**h**), mgbDFC-Mx (**i**) acquired at 0.7 mGy MGD. All images were scaled for maximum detail visibility.

In order to quantitatively compare monochromatic MuCLS mammography with a clinical mammography unit, a contrast-to-noise-ratio (CNR) analysis was performed for a mammographic accreditation phantom (Gammex, Model 156, www.sunnuclear.com/solutions/diagnostic/mammography/156phantom). The image of the accreditation phantom acquired by the conventional mammography system by automated exposure control is compared to both monochromatic absorption-contrast images and also grating-based trimodal images acquired at different mean glandular doses. A CNR analysis for different test objects is presented in Table 2. A selection of images is shown in Fig. 5.

It is clearly visible that the absorption images cevAC-Mx (Fig. 5(a)), mAC-Mx (Fig. 5(b,c)), and mgbAC-Mx (Fig. 5(d)) fulfill the standard criteria of clinical image quality set by the ACR^24^, which require a minimum of 4 fibrils, 3 groups of microcalcifications, and 3 tumor masses to be resolved. A quantitative analysis (Table 2) demonstrates that the CNR in the mAC-Mx (b) image considerably exceeds the CNR in the cevAC-Mx (a) image at equal dose. The mAC-Mx (c) still slightly outperforms the cevAC-Mx image (a) at 20% reduced dose.

For the grating-based images, the mgbAC-Mx (Fig. 5(d)) image at slightly lower dose than cevAC-Mx (a) provides a comparable CNR. Especially for the small tumor masses, the mgbDFC-Mx (f) CNR by far outperforms the clinical image (a), where the smallest tumor mass cannot be distinguished from the background (CNR < 1). This is still the case when the dose is reduced to 35% of the clinical value (i). In addition, the mgbDPC-Mx (e), for which a quantitative analysis is not possible due to the differential nature of the signal, allows to depict all six tumor fibrils. The mgbDFC-Mx (f,i) reveals additional structures within the two largest tumor masses of the accreditation phantom.

Importantly, while the low-dose mgbAC-Mx (g) does not fulfill the ACR criteria, the combination with mgbDPC-Mx (h) and mgbDFC-Mx (i) meets or, in the case of the tumor masses, even exceeds the ACR criteria.

### Resolution

A power spectrum analysis was performed for all absorption and dark-field images cevAC-Mx, mAC-Mx, mgbAC-Mx and mgbDFC-Mx (not for DPC-Mx due to the differential nature of the signal) in order to compare their resolution^25^. As illustrated in Table 3, the resolution is mainly in the range between 3 and 4 linepairs (LP) per mm. The analysis shows that a higher resolution was achieved in the mAC-, mgbAC- and mgbDFC-MX than in the cevAC-Mx for all investigated samples, at lower or equal dose of the monochromatic images.

## Discussion

We presented conventional absorption-contrast as well as grating-based multimodal mammography images of freshly dissected mastectomy samples acquired with a monochromatic x-ray beam at a compact synchrotron source. Comparison with clinical *ex-vivo* mammography images showed equal diagnostic quality at lower dose or superior diagnostic quality at equal dose of the monochromatic images. Tumorous lesions could be identified significantly better in the phase-contrast modality as verified by histopathology (Specimens I, II). Microcalcification clusters were revealed to an equal extent as in the clinical mammography image, at a significantly reduced dose for the monochromatic images (Specimens III, IV). A power spectrum analysis showed that the monochromatic absorption-contrast and dark-field-contrast images (mAC-Mx, mgbAC-Mx, mgbDFC-Mx) could achieve better resolution than the clinical image (cevAC-Mx) at lower or equal dose, proving that the small source size of the MuCLS is beneficial for the resolution.

A dose study conducted with a mammographic accreditation phantom showed a superior CNR of various test objects at equal mean glandular dose and a higher or comparable CNR at 20% reduced dose for the monochromatic mammography images compared to the clinical one. The grating-based phase-contrast and dark-field image yield an enhanced diagnostic content for the fibers and tumor masses, respectively. The dark-field image outperforms the clinical image for the tumor masses even at a 65% lower dose.

The presented results demonstrate that mammography at a compact synchrotron source like the MuCLS has great potential to improve the diagnostic quality of mammography. The compact size and limited financial requirements compared to large-scale synchrotron source allow for envisioning the installation at hospitals in the future. Thus, the implementation of a compact synchrotron could enable a second-level examination in the case of inconclusive diagnosis (as performed at the SYRMEP beamline^4–6^), in addition with the possibility to choose between conventional, but monochromatic absorption imaging and grating-based trimodal imaging.

The main limitation of our study was the different orientation (anteroposterior instead of cranio-caudal or mediolateral-oblique) for three of the four specimens compared to clinical *in-vivo* mammography. The different orientation was necessary in order to avoid artifacts originating from surgical resection margins. Therefore, the *ex-vivo* images are not fully comparable to *in-vivo* images concerning orientation and sample thickness.

The limited sizes of beam and gratings required scanning of the samples, resulting in an increased scan time incompatible with clinical applications. Only recently, the feasibility of manufacturing stitched gratings in order to cover larger fields of view was demonstrated^26^. The field of compact synchrotron sources is constantly developing^22,27,28^ and considerable improvements and further developments of the technology with regard to increased stability, flux and beam size can be expected. An increased field of view can be achieved by employing a larger exit aperture. The currently available 4 mrad opening angle is only limited by the diameter of the exit aperture. By simply replacing the exit aperture by one with a larger diameter, the beam size at the experimental station would increase. In addition, other potential clinical applications will profit especially from an extension of the energy range.

Concerning the mean glandular dose applied in mammography, our results show that dose-compatible grating-based mammography is feasible at a monochromatic source. The used setup even has the potential for further dose reduction by thinning the grating wafers from the currently used 500 μm thick Si substrate down to 200 μm and by modifying the setup to allow positioning of the sample to behind the phase grating.

In conclusion, we believe that monochromatic x-rays provided by compact synchrotron sources like the MuCLS, the first commercially installed source of this type, can enable significant improvements in diagnostic x-ray imaging. Mammography could benefit from enhanced diagnostic image content and improved resolution at a reduced dose. In addition, as the x-ray energy of these sources is tunable, also other applications, like for instance coronary angiography^23^, could benefit from the implementation of compact synchrotron sources in a clinical setting.

## Methods

### Study protocol

The study was conducted in accordance with the Declaration of Helsinki and was approved by the local ethics committee (Ethikkommission of the Ludwig-Maximilian-University, Munich, project number 240-10, date of permission 26/08/2010, amendment 30/05/2012). Inclusion criteria were a histologically proven breast cancer in preoperative core biopsy with a recommendation for mastectomy according to gynecological guidelines or the patient’s wish for mastectomy. Participants gave written informed consent before participation after adequate explanation of the study protocol. Indication for breast surgery followed recommendation of the interdisciplinary tumor board of the University of Munich breast center.

### Preoperative diagnostics

Preoperative diagnostics included clinical breast examination, standard two-view digital mammography in cranio-caudal (CC) and mediolateral-oblique (MLO) projections (Hologic Selenia Dimensions, Bedford, USA) using a standard breast compression paddle and high resolution B-mode ultrasound (standard linear transducer 13.5 MHz, Siemens Acuson Antares, Siemens Healthcare, Germany). Additional MRI was performed in three cases by using a dedicated sensitivity-encoding-enabled bilateral breast coil with a 1.5- or 3.0-Tesla system.

### *Ex-vivo* mammography

The mastectomy samples were intraoperatively marked with surgical sutures for 3D orientation. The samples were fixed within a metal-framed sample holder to afford adequate breast compression. The position was cranio-caudal, or anteroposterior in case a cranio-caudal position was impossible due to the shape of the dissected specimen. A digital *ex-vivo* mammography image was acquired at a clinical mammography unit (Hologic Selenia Dimensions) with a pixel size of 70 × 70 μm^2^.

### Working principle of the MuCLS

**Figure 6:**
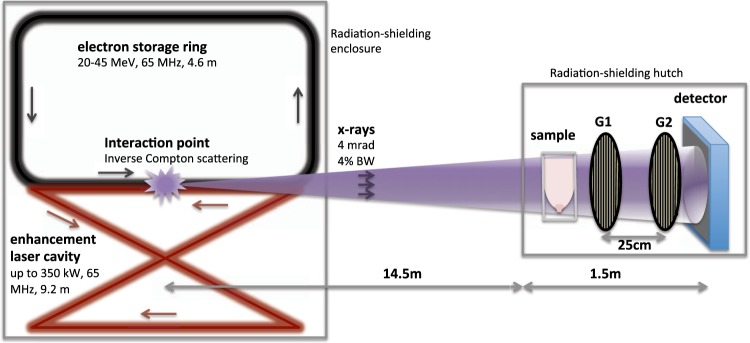
Schematic drawing of the Munich Compact Light Source (MuCLS) and the experimental setup. X-rays are generated in the process of inverse Compton scattering in a laser-electron storage ring design (the linear accelerator section is not drawn here). The grating interferometer is located approximately 16 m from the interaction point. A two-grating interferometer in the first Talbot order was used. The freshly dissected breast specimen is placed in a dedicated sample holder for reasonable sample compression.

The MuCLS is a compact synchrotron based on inverse Compton scattering and was developed and manufactured by Lyncean Technologies Inc., USA. As a relativistic electron bunch collides with a laser pulse, x-ray photons are produced. In good approximation for head-on collision and backscattering, the energy *E*_*x*_ of the emitted x-rays is given by *E*_*x*_ = 4*γ*^2^*E*_*L*_, where *γ* = *E*_*e*_/*E*_0_ with *E*_0_ the rest energy and *E*_*e*_ the total energy of the electron, and *E*_*L*_ is the energy of the laser photons. The electrons circulate in a miniature storage ring and collide with the laser pulse stored in a bow-tie enhancement cavity upon each revolution (cf. Fig. 6)^21,22,29^.

The produced x-rays are collimated to a 4 mrad cone. The x-ray beam is monochromatic, partially coherent, and the energy is tunable between 15 and 35 keV by adjusting the electron energy. The MuCLS currently offers - on a daily basis - a flux exceeding 2.4 · 10^10^ photons per second (at 35 keV), and a source size of less than 50 × 50 μm^2^. At a distance of about 16 m from the interaction point, the beam has an elliptic shape of 62 mm × 74 mm^22^.

### Mammography at the MuCLS

Maintaining the position of the breast specimen within the sample holder in order to ensure comparability, monochromatic mammography images were acquired at the MuCLS. A Talbot interferometer was set up approximately 16 m from the interaction point of the MuCLS, as shown schematically in Fig. 6. The inter-grating distance was ~25 cm, the grating periods were *p*_1_ = 4.9 μm and *p*_2_ = 5.0 μm for phase and analyzer grating, respectively. The signals were retrieved from phase stepping as described in^8^. Conventional absorption-contrast images were acquired without the grating interferometer. A Dexela 1512 flatpanel detector (PerkinElmer Inc., USA) with a Gd_2_O_2_S scintillator was used, yielding an effective pixel size of 71 × 71 μm^2^.

Scanning the sample and afterwards stitching of several images was required to cover the whole sample, using linear ramps to blend the overlapping areas. Table 4 summarizes the acquisition parameters for each scan. The exposure times in Table 4 are given for one Stitching-scan. The MuCLS x-ray energy was tuned to 25 keV for all samples. The interferometer visibility was approximately 45–50% for all measurements.

### Dose calculation

The MGD automatically registered by the clinical mammography unit for clinical *in vivo*- and *ex vivo*-images is given in Table 4 for each sample. It is calculated based on the x-ray tube settings and the compressed sample thickness. The MGD value for the cevAC-Mx images was corrected when an incorrect sample thickness was assumed due to the shape of the sample holder. A correction factor was calculated based on the half value layer of Aluminum for the respective acquisition settings and the assumed and correct breast thickness, from tabulated values by^30,31^.

For the monochromatic mammography performed at the MuCLS, the MGD was calculated using monoenergetic normalized glandular dose coefficients DgN(*E*) (with *E* the x-ray energy) as tabulated by Boone *et al*.^32^, taking into account the MuCLS spectrum^22^, and summing over all energy bins *E*:

1 $${\rm{M}}{\rm{G}}{\rm{D}}=\sum _{E}\,K(E)[{\rm{m}}{\rm{G}}{\rm{y}}]\cdot 0.114\,[\frac{{\rm{R}}}{{\rm{m}}{\rm{G}}{\rm{y}}}]\cdot ({\rm{D}}{\rm{g}}{\rm{N}}(E)\,[\frac{{\rm{m}}{\rm{G}}{\rm{y}}}{{\rm{R}}}]).$$

The formula for the MGD was adapted from^32^ to be used with air kerma *K* instead of the older unit exposure, as the fitted equation for exposure given by^32^ is incorrect^33^. The DgN (*E*) values were selected according to the compressed thickness of the dissected breasts, and assuming a 50%/50% distribution of glandular and adipose tissue. The air kerma for the MuCLS beam *K* per energy bin *E* can be calculated for known photon flux Φ at the sample position and x-ray spectrum^34^:

2 $$K(E)=E\cdot {\rm{\Phi }}(E)\cdot {({\mu }_{{\rm{en}}}/\rho )}_{{\rm{air}}}(E),$$

where (*μ*_en_/*ρ*)_air_ (*E*) is the mass energy attenuation coefficient of air^35^. The photon flux per energy bin Φ(*E*) was calculated from a single photon counting Pilatus 200 K detector (Dectris Ltd., Switzerland), taking into account the x-ray spectrum and the efficiency of the Si sensor of the detector^36^. The calculation of the air kerma was validated using a soft x-ray ionization chamber (Model 34013, PTW Freiburg GmbH, Germany) and values agreed within the measurement uncertainties of ±10%. A scintillation counter was calibrated to calculated air kerma prior to each sample measurement and the scintillation counts were logged for each measurement frame, thus yielding an exact measure of air kerma for each scan.

### Image Analysis

The CNR was calculated according to the definition

3 $${\rm{CNR}}=\frac{\overline{{S}_{1}}-\overline{{S}_{2}}}{{\sigma }_{{\rm{BG}}}},$$

where $\overline{{S}_{1}}$ and $\overline{{S}_{2}}$ are the average signals in two regions of interest (ROI) which should be compared, and *σ*_BG_ is the standard deviation within a larger ROI located in the background region.

The resolution of the images was determined by analyzing their power spectra^25^. The images were Fourier transformed and a Gaussian filter was applied on the squared norm. The resolution of the images is then given by the maximal spatial frequency where the spectral power of the signal equals twice the spectral power of the noise baseline, taking into account the effective pixel size of clinical and experimental images.
